# Anti-inflammatory, Antinociceptive, and Antioxidant Activities of Methanol and Aqueous Extracts of *Anacyclus pyrethrum* Roots

**DOI:** 10.3389/fphar.2017.00598

**Published:** 2017-09-05

**Authors:** Houria Manouze, Otmane Bouchatta, A. Chemseddoha Gadhi, Mohammed Bennis, Zahra Sokar, Saadia Ba-M’hamed

**Affiliations:** ^1^Laboratory of Pharmacology, Neurobiology and Behavior (URAC-37), Faculty of Sciences Semlalia, Cadi Ayyad University Marrakech, Morocco; ^2^Unit of Phytochemistry and Pharmacology of Aromatic and Medicinal Plants, Laboratory of Biotechnology, Protection and Valorization of Plant Resources (URAC35), Faculty of Sciences Semlalia, Cadi Ayyad University Marrakech, Morocco

**Keywords:** *Anacyclus pyrethrum*, anti-inflammatory, antinociceptive, antioxidant, mice

## Abstract

*Anacyclus pyrethrum* (L.) is a plant widely used in Moroccan traditional medicine to treat inflammatory and painful diseases. The objective of the present study was to evaluate the antinociceptive, anti-inflammatory and antioxidant activities of aqueous and methanol extracts of *Anacyclus pyrethrum* roots (AEAPR and MEAPR). The anti-inflammatory effect of AEAPR and MEAPR was determined in xylene–induced ear edema and Complete Freund’s Adjuvant (CFA)-induced paw edema. The antinociceptive activity of AEAPR and MEAPR (125, 250, and 500 mg/kg) administered by gavage was examined in mice by using acetic acid-induced writhing, hot plate, and formalin tests, and the mechanical allodynia were assessed in CFA-induced paw edema. In addition, the *in vitro* antioxidant activities of the extracts were determined by using 2,2-diphenyl-1-picrylhydrazyl (DPPH) radical scavenging method, ferric reducing power and β-carotene-linoleic acid assay systems. AEAPR and MEAPR produced significant reductions in CFA-induced paw edema and xylene-induced ear edema. A single oral administration of these extracts at 250 and 500 mg/kg significantly reduced mechanical hypersensitivity induced by CFA, which had begun 1 h 30 after the treatment, and was maintained till 7 h. Chronic treatment with both extracts significantly reduced mechanical hypersensitivity in persistent pain conditions induced by CFA. Acute pretreatment with AEAPR or MEAPR at high dose caused a significant decrease in the number of abdominal writhes induced by acetic acid injection (52.2 and 56.7%, respectively), a marked increase of the paw withdrawal latency in the hot plate test, and also a significant inhibition of both phases of the formalin test. This antinociceptive effect was partially reversed by naloxone pretreatment in the hot plate and formalin tests. Additionally, a significant scavenging activity in DPPH, reducing power and protection capacity of β-carotene was observed in testing antioxidant assays. The present study suggests that AEAPR and MEAPR possess potent anti-inflammatory, antinociceptive and antioxidant effects which could be related to the presence of alkaloids and phenols in the plant. In addition, the antinociceptive effect of APR extracts seems to partly involve the opioid system. Taken together, these results suggest that *Anacylcus pyrethrum* may indeed be useful in the treatment of pain and inflammatory disorders in humans.

## Introduction

Chronic pain is a serious problem globally. Pain affects one in five adults, while an estimated one in ten suffers from chronic pain each year (Goldberg and McGee, 2011). Because of their involvement in most diseases, inflammation and pain have become the most important topic of several scientific researches. Although non-steroidal anti-inflammatory drugs (NSAIDs) and opiates have been classically used to manage pain, some adverse reactions may occur with these drugs, such as gastrointestinal disturbances, renal damage, respiratory depression, and possible dependence (Burke et al., 2006; Farshchi et al., 2009; Vittalrao et al., 2011). Therefore, new and more effective anti-inflammatory and analgesic drugs without side effects are targeted as an alternative to NSAIDs and opiates (Kumara, 2001; Dharmasiri et al., 2003). Recently, research has focused on herbal medicines used in traditional medicine due to their low cost, efficacy and safety. According to the World Health Organization (WHO), about 80% of the world population still relies mainly on herbal remedies (Kumara, 2001; Li et al., 2003).

*Anacylcus pyrethrum* (L.) Link (Asteraceae) is a native plant of North Africa (Boulos, 1983), locally known as “Aqar-qarha” or “Tigandizt” by Moroccan people. The root is widely used in Moroccan traditional medicine to treat rheumatism, sciatica, colds, neuralgia, and paralysis (Bellakhdar, 1997). It is also considered to be a sialagogue, sudorific and to relieve toothache (Doudach et al., 2012; Zaidi et al., 2013). Previous studies have indicated that the plant possesses antimicrobial activities (Doudach et al., 2012; Jalayer et al., 2012; Selles et al., 2013), and it has anti-diabetic (Tyagi et al., 2011; Selles et al., 2012b), aphrodisiac (Vikas et al., 2009) and hepatoprotective effects (Usmani et al., 2016). The plant is reported to have immune-modulatory and immune-stimulating properties (Bendjeddou et al., 2003; Ching et al., 2007; Dalila et al., 2010), in addition to anti-inflammatory and antioxidant potential (Müller et al., 1993; Sujith et al., 2011a; Selles et al., 2012a).

The phytochemical screening of *Anacylcus pyrethrum* has led to the identification of various secondary metabolites such as alkaloids, reducing compounds, tannins, flavonoids and coumarins (Hanane et al., 2014). This species also contains saponins, sesamin, inulin, gum and traces of essential oil (Sujith et al., 2011b; Selles et al., 2012b). The most important phytoconstituents present in its root are N-isobutyldienediynamide and polysaccharides (Crombie, 1954; Bendjeddou et al., 2003; De Spiegeleer et al., 2011; Boonen et al., 2012).

Although some *Anacyclus* species such as *Anacyclus clavatus* have been studied for their anti-inflammatory and antioxidant activities (Aliboudhar and Tigrine-Kordjani, 2014), no scientific reports on the antinociceptive or anti-inflammatory activities of *Anacyclus pyrethrum in vivo* have been conducted. Thus, the aim of the present study was to provide scientific evidence for the antinociceptive and anti-inflammatory activities of methanol and aqueous extracts of *Anacyclus pyrethrum* roots (APR) using appropriate models in mice. In addition, as inflammation is a process linked to oxidative stress and to the over-production of the reactive oxygen species (ROS), the antioxidant capacity of both extracts was also evaluated.

## Materials and Methods

### Preparation of the Extracts and Phytochemical Screening

#### Plant Material

*Anacylcus pyrethrum* (L.) Link (Asteraceae) was collected in June 2014, from Oukaïmeden (74 km from Marrakech) at 2,600 m of altitude in the High-Atlas Mountains (Morocco). The plant was identified by Professor A. Ouhammou, a taxonomist in the department of Biology, Faculty of Sciences Semlalia, Cadi Ayyad University. A voucher specimen was deposited at the Faculty’s Herbarium (Mark 8258).

#### Extracts Preparation

The roots were separated from the aerial parts of the plant and dried under shade. They were ground to a fine powder using a grinder apparatus. Root powder (1 g) was stirred with distilled water (20 ml) for 24 h at room temperature. The aqueous macerate was centrifuged (1200 rpm) for 15 min. The supernatant was lyophilized (yield = 20% w/w) then stored in a freezer at -20°C until experimental use. For methanol extract preparation, the powder of APR (400 g) was exhaustively extracted with methanol in a Soxhlet apparatus. The methanol extract was concentrated to dryness under vacuum. The residue (21.8% w/w) was stored at -20°C for several months, until the experimental use.

#### Phytochemical Screening of APR Extracts

Aqueous and methanol extracts of APR were screened for the presence of flavonoids, alkaloids, terpenoids, tannins and saponins. The qualitative determination of these phytochemicals was conducted using previously reported methods (Harborne, 1998; Bargah, 2015).

1. Test for flavonoids: 2 ml of APR extract was evaporated and the residue was taken up in 5 ml of alcohol (50%) and 1 ml of concentrated hydrochloric acid. After addition of a few magnesium chips, the presence of flavonoids was indicated by the apparition of a red color.
2. Test for terpenoids: 1 mg of APR extract was dissolved in a few drops of acetic acid in 3 ml of the mixture (acetic anhydride-concentrated sulfuric acid 50:1 v/v). The development of a green color indicated the presence of terpenoids.
3. Test for tannins: APR extract (5 mg) was dissolved in 20 ml of distilled water and heated to boiling on a hot plate. Tannins were detected by the apparition of a green color after the addition of a few drops of an aqueous solution of ferric chloride (FeCl_3_; 9%).
4. Test for saponins: APR extract (500 mg) was dissolved in 10 ml of distilled water in a test tube. The tube was shaken vigorously for 15 s and then allowed to stand for 15 min. The presence of stable foam indicated the presence of saponins.
5. Test for alkaloids: APR extract (500 mg) was stirred in 50 ml of sulfuric acid (0.1 N) for 15 min. After filtration, a concentrated ammonia solution (5 ml) was added to the solution. The alkaloids were then extracted with 50 ml of dichloromethane. After evaporation under *vacuum* of the organic layer, the residue was dissolved in 1 ml of methanol. A few drops of Dragendorff reagent were added to the solution; the formation of a precipitate was taken as a hint for the presence of the alkaloids.

### Animals

Adult Swiss male mice (25–35 g) were used for *in vivo* bioassays. The animals were provided by the Animal Care Facility of the Faculty of Sciences Semlalia, Cadi Ayyad University, Marrakech, Morocco. The mice were kept under constant conditions of ambient temperature (22 ± 2°C) under a 12 h light/12 h dark cycle, with *ad libitum* access to food and water. All animal procedures were in strict accordance with the guidelines of the European Council Directive (EU2010/63). Care was taken to minimize the number of animals used for the experiments. All efforts were made to minimize any animal suffering, and the study met the ethical standards and approvals of the Council Committee of the research laboratories of the Faculty of Sciences, Cadi Ayyad University of Marrakech.

### Drugs and Reagents

Indomethacin was purchased from Laprophan (Morroco) and naloxone from Hospira (United States). Acetic acid, formalin, xylene, xylazine, ketamine and CFA (suspension of heat-killed Mycobacterium tuberculosis in oil, 1 mg/ml) were obtained from Sigma–Aldrich (France).

### Acute Toxicity

The acute toxicity study was conducted according to the Organization for Economic Cooperation and Development (OECD) guideline no. 423 (OECD, 2001), where the limit test dose of 5000 mg/kg was used. Mice were divided equally into nine groups (six animals per group). Eight groups were orally treated by gavage with different doses (500, 1000, 2000, and 5000 mg/kg) of AEAPR or MEAPR solution at 10 ml/kg. One group that received vehicle (distilled water) was included as a negative control. To detect signs of toxicity and death, mice were observed within the first 12 h after drug administration. The mice were daily weighed and observed for 14 days after treatment. At the end of the 14-day period, the animals were injected with a urethane lethal dose (1 g/kg, i.p.) and the vital organs were immediately removed and weighed.

### Assessment of APR Extracts Anti-inflammatory Activity

#### Xylene-Induced Ear Edema

The xylene-induced ear edema test was performed as previously described (Tang et al., 1984). Briefly, each mouse was given by gavage one dose (125, 250, or 500 mg/kg) of AEAPR or MEAPR, indomethacin (10 mg/kg) or vehicle (10 ml/kg) 1 h before induction of ear edema by topical application of 0.02 ml xylene on the inner and outer surfaces of the right ear. The left ear was used as a control. One hour after xylene application, mice (six animals per group) were sacrificed by cervical dislocation. Circular sections of 5 mm were excised and weighed. To evaluate the extent of the ear edema, we calculated the weight difference between the right and the left ear sections of the same animal.

#### Complete Freund’s Adjuvant-Induced Paw Edema

Eight groups of animals (six mice per group) were assigned to this test. Mice were anesthetized with a mixture of ketamine (50 mg/kg) and xylazine (2 mg/kg) cocktail. All groups, except vehicle control group, received 20 μl of CFA by a subcutaneous injection in the plantar surface of the right hind paw (Bortalanza et al., 2002). Twenty four hours after CFA injection, mice were treated by gavage with AEAPR, MEAPR (125, 250, or 500 mg/kg), indomethacin (10 mg/kg), or vehicle (10 ml/kg). The effect of each treatment on edema development induced by the intraplantar injection of CFA was evaluated according to the previously reported method of Milano et al. (2008). The level of inflammation of the right hind paw was measured using a caliper at several time-points (0, 0.5, 1, 2, 4, 5, 6, and 7 h) and was expressed in millimeters.

### Assessment of APR Extracts Antinociceptive Activity

#### Mechanical Allodynia

To measure the nociceptive reactivity to the application of mechanical stimuli to the hind pad, each mouse was placed in an individual observation cage (12 cm × 12 cm × 12 cm) with a mesh floor allowing access to the ventral surface of the hind pads. The animals were accustomed to the cage and the experiment room for at least 10–15 min or at the end of the exploratory behavior. Then mechanical hypersensitivity was assessed as described by Chaplan et al. (1994). Calibrated Von Frey filaments of increased tension were applied through the wire mesh floor of the cage, 10 times per paw with enough force to cause buckling of the filament. The withdrawal threshold for Von Frey assay was determined as the filament at which the animal withdrew its paw at least five times in ten applications. Basal responsiveness to mechanical stimuli was assessed on the day before CFA injection. Twenty-four hours after CFA injection, mice were treated by gavage with AEAPR, MEAPR (125, 250, or 500 mg/kg), indomethacin (10 mg/kg), or vehicle (10 ml/kg) and then, the withdrawal threshold was evaluated at several time points, but always between 9 am and 6 pm.

To investigate the effect of chronic treatment on paw withdrawal, and the possible development of tolerance, mice (six animals per group) were treated with AEAPR, MEAPR (125, 250, or 500 mg/kg), indomethacin (10 mg/kg), or vehicle (10 ml/kg) once a day for 5 days successively. In order to investigate the possible development of tolerance, the treatment was interrupted for 3 days (from day 6 to day 8) and reinitiated for 2 days again (day 9 and day 10). The mechanical hypersensitivity was assessed 3 h after each daily treatment (the time where the maximal response was observed in the acute treatment). For the days 6–8 (without treatment), the test was performed at exactly the same time as the previous days.

#### Thermal Hyper-Nociception

In this test, animals were individually placed on a hot plate with an adjustable temperature (to 55 ± 1°C) (Bhandare et al., 2010). The reaction time was defined as the latency for the animal to lick its paw(s) or jump from the plate. The cutoff time for the hot plate latencies was set at 30 s. Ten groups of six mice each were used for this test. They were treated orally with 125, 250, or 500 mg/kg of AEAPR or MEAPR, vehicle (10 ml/kg), indomethacin (10 mg/kg), naloxone (1 mg/kg) and AEAPR (500 mg/kg) or naloxone (1 mg/kg) and MEAPR (500 mg/kg). Naloxone, an opioid receptor antagonist, was administered alone or 30 min before the administration of MEAPR or AEAPR. The latency of nociceptive response was recorded before treatment and at 30, 60, 90, 120, 150, 180, 210, and 240 min after drug administration.

#### Acetic Acid-Induced Abdominal Writhing

This test was performed in mice, according to the method described by Ferreira et al. (2000). Briefly, 0.6% acetic acid solution (10 ml/kg) was injected intraperitoneally. Animals (six mice per group) were pre-treated with AEAPR or MEAPR (125, 250, or 500 mg/kg), vehicle (10 ml/kg) or indomethacin (10 mg/kg), 30 min prior to peritoneal irritation. The resulting writhes and stretching were observed and counted over a period of 60 min after acetic acid injection.

#### Formalin-Induced Pain

Formalin test was carried out as previously reported (De Miranda et al., 2001). Ten groups of six mice each were used for this test: the negative control group (vehicle, 10 ml/kg), the positive control group (indomethacin, 10 mg/kg), six groups receiving AEAPR or MEAPR (125, 250, or 500 mg/kg) and three groups treated with naloxone (1 mg/kg): alone, 30 min before the administration of MEAPR or AEPRR (500 mg/kg). After treatment administration, each animal received an intraplantar injection of 2% formalin (20 μl/ animal) into the right paw. Total time spent licking the injected paw was recorded during two phases: 5–10 min after formalin injection and 15–30 min after formalin injection (Hunskaar and Hole, 1987; Tjølsen et al., 1992).

### Motor Performance and Locomotor Activity

To evaluate any coordination disruption, non-specific muscle-relaxant or sedative effects of APR extracts, mice were subjected to the rotarod task and open-field test. The motor coordination of the mice was evaluated on the rotarod apparatus at a constant speed of 12 rotations per minute. Twenty-four hours prior the drug testing, animals were tested and those who remained at the rotating bar for the full 300 s during three consecutive trials were used for the subsequent experiments. The selected animals were randomly distributed into groups of six mice and received AEAPR or MEAPR orally at a dose of 125, 250, or 500 mg/kg. The control group received the same volume of vehicle (10 ml/kg). An additional group received indomethacin (10 mg/kg) and served as a positive control. Rotarod tests were performed prior to drug administration (basal) and at 30, 60, and 120 min after administration. Latency to fall off was measured for each session (up to 300 s).

In order to evaluate eventual motor impairment induced by plant extract, the mice were placed individually in an observation chamber 60 min after oral treatment with vehicle, indomethacin or APR extracts. The open field apparatus used was a 50 × 50 cm square arena with 30 cm high black walls. The animal was placed in the center of the arena and distance traveled was scored during 10 min, using the videotracking EthoVision XT8.5 software (Noldus, Netherlands). The animal was returned to its home cage, and the apparatus cleaned with ethanol 70% to remove any odor.

### Antioxidant Activity

#### DPPH Free Radical-Scavenging Activity

The hydrogen or electron donation ability of APR extracts (MEAPR, AEAPR) was measured using the stable radical 1, 1diphenyl-2-picryl hydrazyl (DPPH) assay (Burits and Bucar, 2000). Various concentrations of MEAPR and AEAPR were prepared in methanol and methanol-water, respectively (V/V). An aliquot (2 ml) of a 60 μM methanol solution of DPPH was mixed with 50 μl solution of each sample. After 20 min incubation at room temperature, the absorbance was read at 517 nm. DPPH free radical-scavenging activity of each sample was expressed as the percentage decrease of DPPH absorbance versus control. It was calculated using the formula: % Inhibition = [(A_b_ – A_a_)/A_b_] × 100, where A_b_ is the absorbance of DPPH alone in methanol (control) and A_a_ is the absorbance of DPPH in the presence of the test substance. Quercetin and butylated-hydroxyl-toluene (BHT) were used as positive controls.

#### β-Carotene/Linoleic Acid Bleaching Assay

The β-carotene/linoleic acid test evaluates the lipoperoxydation inhibitory effect of a compound or a mixture of compounds. The method described by Miraliakbari and Shahidi (2008), was used with slight modifications. A mixture of β-carotene and linoleic acid was prepared by adding together 0.5 mg β-carotene in 1 ml chloroform (HPLC grade), 25 μl linoleic acid and 200 mg Tween 40. The chloroform was then completely evaporated under vacuum and 100 ml of oxygenated distilled water was subsequently added to the residue and mixed to form a clear yellowish emulsion. Three hundred fifty microliters of various concentrations of the sample (MEAPR, AEAPR, BHT or Quercetin) were added to 2.5 ml of the above emulsion in test tubes and mixed. The test tubes were incubated in a water bath at 50°C for 2 h together with a negative control (blank) containing methanol instead of samples. The absorbance values were measured at 470 nm. The lipoperoxydation inhibitory effect of testing samples was evaluated by calculating the inhibition percentage using the following equation: I% = (A_β-caroteneafter2_ _hassay_/A_initial_ _β-carotene_) × 100, where A_β-carotene_ _after2_ _hassay_ is the absorbance of β-carotene remaining in the sample after 2 h and A_initial_ _β-carotene_ is the absorbance of β-carotene at the beginning of the experiment.

#### Reducing Power Determination

The ability of APR extracts to reduce Fe^+3^ to Fe^+2^ was investigated using the method of Oyaizu (1986). The tested sample (MEAPR, AEAPR or control) was mixed with phosphate buffer (2.5 ml, 0.2 M, pH 6.6) and potassium ferricyanide (2.5 ml, 1%). The mixture was then incubated at 50°C for 20 min. Subsequently, 2.5 ml of trichloroacetic acid (10%) was added to the mixture, which was then centrifuged for 10 min at 3000 rpm. Finally, the upper layer of the solution (2.5 ml) was mixed with distilled water (2.5 ml) and FeCl_3_ (0.5 ml, 0.1%), and the absorbance was measured at 700 nm in a spectrophotometer. BHT and Quercetin were used as positive controls.

### Statistical Analysis

Statistical analysis was performed using SigmaPlot 11.0 software. All data were presented as mean ± SEM for six mice per group. A two-way analysis of variance (ANOVA) with repeated measures followed by Holm–Sidak *post hoc* analysis was used to examine the time-courses of mechanical, thermal and anti-edema effects after various treatments. Kruskal–Wallis ANOVA or one-way ANOVA followed by *post hoc* testing with the Student-Newman–Keuls test for multiple comparisons were used to measure variance of mouse behavior between groups. The significance of the differences between the means in the antioxidant activity was assessed by Student’s *t*-test. The significance threshold was set at *p* < 0.05.

## Results

### Phytochemical Screening of APR Extracts

The qualitative phytochemical screening of AEAPR and MEAPR showed the presence of alkaloids, flavonoids, tannins, saponins, and terpenoids in both extracts.

### Acute Toxicity

The oral administration of AEAPR or MEAPR at doses up to 5000 mg/kg did not produce any visible signs or symptoms of toxicity in mice. No mortality and no significant changes in body weights (*p* > 0.05) or in organ weights (*p* > 0.05) were observed at 14 days after AEAPR or MEAPR administration (**Table 1**).

**Table 1:** Effect of a single oral administration of AEAPR and MEAPR on body weight and relative organ weights of mice.

	Control	AEAPR (mg/kg)				MEAPR (mg/kg)			
		500	1000	2000	5000	500	1000	2000	5000
Body weight (g)	28.83 ± 0.56	28.52 ± 1.16	28.36 ± 0.75	27.86 ± 1.30	27.72 ± 0.66	28.48 ± 1.58	28.47 ± 1.14	27.70 ± 1.16	27.48 ± 0.69
**Relative organ weights (g/ 100 b.wt)**									
Brain	1.71 ± 0.10	1.69 ± 0.08	1.70 ± 0.07	1.71 ± 0.11	1.71 ± 0.10	1.69 ± 0.08	1.70 ± 0.04	1.71 ± 0.05	1.71 ± 0.14
Kidney	0.82 ± 0.04	0.81 ± 0.05	0.82 ± 0.04	0.82 ± 0.17	0.82 ± 0.15	0.82 ± 0.08	0.82 ± 0.04	0.82 ± 0.09	0.83 ± 0.15
Liver	6.48 ± 0.60	6.55 ± 0.65	6.46 ± 0.76	6.94 ± 0.94	6.49 ± 0.53	6.58 ± 0.70	6.67 ± 0.42	6.90 ± 0.34	6.95 ± 0.56

*Data are expressed as mean ± SEM. There were no statistically significant differences in the body weight (b.wt) or relative organ weights*.

### Anti-inflammatory Effect of *Anacyclus pyrethrum root* Extracts

#### Xylene-Induced Ear Edema

**FIGURE 1:**
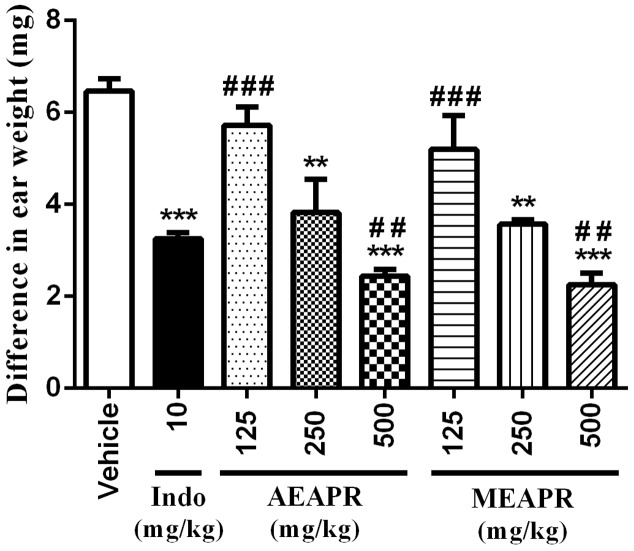
Effect of AEAPR and MEAPR administrated orally on xylene-induced mouse ear edema test. Animals (six mice each group) were treated orally with distilled water (vehicle), indomethacin (10 mg/kg: Indo), MEAPR or AEAPR (125, 250, and 500 mg/kg), prior to topical application of xylene (0.02 ml) on the right ear of the mice. Values are mean ± SEM of weight difference (in mg) between the right and the left ear sections of the same animal. ^∗∗^*p* < 0.01 and ^∗∗∗^*p* < 0.001 compared to vehicle-treated group; ^##^*p* < 0.01 and ^###^*p* < 0.001 compared to CFA + indomethacin-treated group, using Kruskal–Wallis ANOVA test.

Topical application of xylene caused a significant increase in the right ear section’s weight (11.68 ± 0.49 mg) when compared to the vehicle-treated left ear (5.22 ± 0.29 mg) (*p* < 0.001). Indomethacin, as well as both APR extracts at tested doses, reduced xylene-induced ear edema compared to the vehicle-treated control group (**Figure 1**). Indeed, Kruskal–Wallis one-way analysis of variance confirmed a high significant difference between all groups [*H*(7) = 30.63, *p* < 0.001]. The *post hoc* analysis showed that the pretreated groups with APR extracts or indomethacin decreased significantly (*p* < 0.001) the edema induced by xylene in comparison with vehicle-treated group. In the group treated with indomethacin, the inhibition of ear edema induced by xylene was significantly higher than that of the group treated with 125 mg/kg of MEAPR or AEAPR (*q* = 4.61 and *q* = 4.77, *p* < 0.001 respectively), whereas the oral administration of the two extracts at a dose of 500 mg/kg exhibited a very strong anti-edema effect compared to the indomethacin group (*q* = 5.05 and *q* = 6.46, *p* < 0.001; respectively) (**Figure 1**). However, we observed no significant difference in ear edema between AEAPR and MEAPR at a dose of 250 mg/kg vs. indomethacin (*q* = 2.73 and *q* = 1.95, *p* > 0.05) nor between AEAPR vs. MEAPR groups (*q* = 1.03, *p* > 0.05). The positive control, indomethacin, inhibited edema by 49%, whereas MEAPR and AEAPR at a dose of 500 mg/kg inhibited it respectively by 65% and 62% compared to the group treated with the vehicle.

#### CFA-Induced Inflammatory: Paw Thickness

**FIGURE 2:**
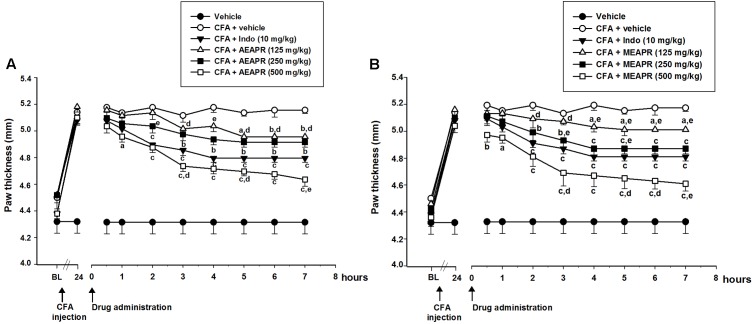
Effect of acute administration of AEAPR **(A)** and AEAPR **(B)** on CFA-induced paw edema. Animals (six mice each group) were treated orally with vehicle (distilled water), indomethacin (Indo), MEAPR or AEAPR (125, 250, and 500 mg/kg), 24 h after intraplantar injection of Complete Freund’s adjuvant (CFA: 20 μl). Each point represents the mean ± SEM of the paw thickness (in mm), before (basal level: BL), 24 h after the injection of CFA (time point 24 h) and at the time points indicated after drug administration. Holm–Sidak *post hoc* analysis; ^a^*p* < 0.05, ^b^*p* < 0.01 and ^c^*p* < 0.001 in comparison with CFA + vehicle treated group; ^d^*p* < 0.05; ^e^*p* < 0.01 in comparison with CFA + indomethacin treated group, using Holm–Sidak *post hoc* analysis.

Complete Freund’s Adjuvant injection caused a significant increase (*t* = 10.59, *p* < 0.001) in paw volume after 24 h (from 4.50 ± 0.08 mm to 5.10 ± 0.05 mm). The paw thickness was reduced respectively by MEAPR, AEAPR and indomethacin after 30, 60, and 120 min of oral administration (5.04 ± 0.00 mm, 4.94 ± 0.04 mm and 4.90 ± 0.03 mm, respectively) (**Figure 2**). This reduction was statistically different between experimental groups [*F*_(8,54)_ = 23.49, *p* < 0.001], and also between different post-treatments’ times [*F*_(8,54)_ = 399.38, *p* < 0.001]. Both APR extracts showed dose- and time-dependent anti-inflammatory effects. At higher doses (250 and 500 mg/kg), this effect became significant, starting from the third hour after AEAPR administration (**Figure 2A**) and starting from the second hour for MEAPR (**Figure 2B**). It does not become significant until the fourth and fifth hour after the administration of the low dose (125 mg/kg) of MEAPR or AEAPR (*t* = 2.16 and *t* = 2.39, *p* < 0.05, respectively). In addition, at a higher dose, AEAPR and MEAPR exhibited a greater reduction effect in paw thickness than indomethacin from time points 3 h (*t* = 2.50 and *t* = 2.69, *p* < 0.05; respectively), to 7 h (*t* = 3.10 and *t* = 3.59, *p* < 0.01; respectively).

### Antinociceptive Effect of *Anacyclus pyrethrum* Root Extracts

#### CFA-Induced Mechanical Hypersensitivity

**FIGURE 3:**
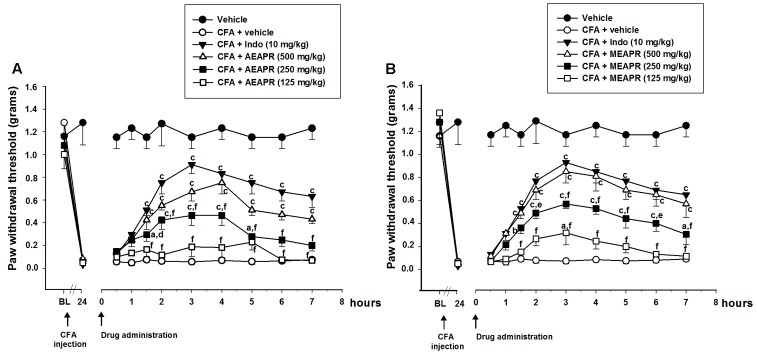
Effect of acute administration of AEAPR **(A)** and MEAPR **(B)** on mechanical hypersensitivity in mice. Animals (six mice each group) were treated orally with vehicle (distilled water), indomethacin (Indo), MEAPR or AEAPR (125, 250, and 500 mg/kg), 24 h after intraplantar injection of Complete Freund’s adjuvant (CFA; 20 μl). Each point represents the mean ± SEM of the paw withdrawal threshold (in grams) to hind paw stimulation, before (basal level: BL), 24 h after the injection of CFA (time 24 h) and at the times indicated after drug administration. ^a^*p* < 0.05, ^b^*p* < 0.01 and ^c^*p* < 0.001 in comparison with CFA + vehicle treated group; ^d^*p* < 0.05; ^e^*p* < 0.01 and ^f^*p* < 0.001 in comparison with CFA + indomethacin treated group, using Holm–Sidak *post hoc* analysis.

The intraplantar injection of CFA produced noticeable mechanical hypersensitivity (The paw withdrawal threshold reduced from 1.57 ± 0.15 g at baseline to 0.42 ± 0.04 g (*t* = 12.21; *p* < 0.001), 24 h after CFA injection (**Figure 3**). The single oral administration of AEAPR or MEAPR, at 250 and 500 mg/kg, reduced the mechanical hypersensitivity induced by CFA. The paw withdrawal threshold increased significantly from point time 1 h 30 to 3 h and remained high up to 7 h (**Figure 3**). Two-way repeated measures ANOVA revealed that the withdrawal threshold was significantly different between groups [*F*_(8,54)_ = 51.89, *p* < 0.001] and varied significantly with time [*F*_(8,54)_ = 178.95, *p* < 0.001]. The *post*-*hoc* analysis showed a significant increase of withdrawal threshold at time point of 1h30 in mice treated with AEAPR or MEAPR (250 and 500 mg/kg) and indomethacin compared to the CFA group (AEAPR: *t* = 2.22, *p* < 0.05 and *t* = 3.50, *p* < 0.001; MEAPR: *t* = 2.70, *p* < 0.01 and *t* = 3.98, *p* < 0.001; indomethacin: *t* = 4.38, *p* < 0.001; respectively). The CFA group did no differ at 125 mg/kg of both extracts, except for MEAPR at the time point 3 h (*t* = 2.23, *p* < 0.05). Moreover, no significant difference was observed between indomethacin and MEAPR or AEAPR at 500 mg/kg (*t* = 0.40 and *t* = 0.88, *p* > 0.05).

**FIGURE 4:**
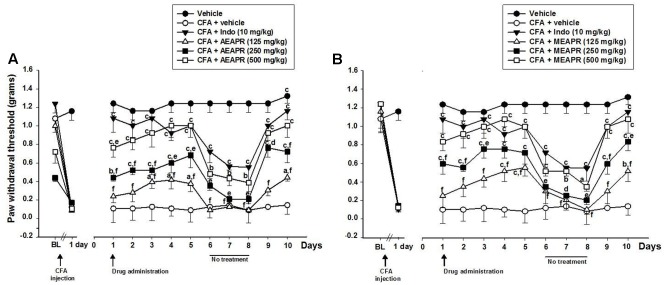
Effect of chronic administration of AEAPR **(A)** or MEAPR **(B)** on mechanical hypersensitivity in mice. Animals (six mice each group) were treated orally with vehicle (distilled water), indomethacin (Indo), MEAPR or AEAPR (125, 250, and 500 mg/kg), 24 h after intraplantar injection of Complete Freund’s adjuvant (CFA; 20 μl). Each point represents the mean ± SEM of the paw withdrawal threshold (in grams) to hind paw stimulation before (basal level: BL), 24 h after CFA injection and at the times (days) indicated after drug administration. The treatment was given once a day for 5 days successively. On the 6th, 7th, and 8th day the drug treatment was avoided and reinitiated for 2 days again in order to evaluate resistance effect of the APR extracts. ^a^*p* < 0.05, ^b^*p* < 0.01 and ^c^*p* < 0.001 in comparison with CFA + vehicle treated group; ^d^*p* < 0.05; ^e^*p* < 0.01 and ^f^*p* < 0.001 in comparison with CFA + indomethacin treated group, using Holm–Sidak *post hoc* analysis.

To investigate the effects of repeated treatment, mice received daily MEAPR or AEAPR (125, 250, or 500 mg/kg), indomethacin, or vehicle for 5 days, interrupted for 3 days, then given daily for 2 more days (**Figure 4**). The results demonstrated that the threshold responses of animals after AEAPR or MEAPR treatments were increased during the observation period compared to CFA group. When the treatments were interrupted for 3 days, mechanical allodynia was re-established. On the 9th day, the treatments were restarted and it was observed that both extracts of APR once again reduced mechanical allodynia.

A two-way repeated measures ANOVA (treatment and day) revealed a significant effect of treatment [*F*_(8,54)_ = 171.13, *p* < 0.001] and time [*F*_(8,54)_ = 60.97, *p* < 0.001]. On the first day of treatment (day 1), except for the dose of 125 mg/kg of both extracts, the *post hoc* analysis showed significant differences between CFA group and MEAPR (250 and 500 mg/kg; *t* = 4.114 and *t* = 6.098, *p* < 0.001; respectively), AEAPR (250 and 500 mg/kg; *t* = 2.775, *p* < 0.01 and *t* = 5.433, *p* < 0.001; respectively) and indomethacin (*t* = 8.091, *p* < 0.001) treated groups. In addition, there was no significant difference, neither between MEAPR and AEAPR groups at 500 mg/kg nor between MEAPR and indomethacin groups (*p* > 0.05), whereas a significant difference between AEAPR and indomethacin groups (*t* = 2.66, *p* < 0.01) was shown. During the first treatment period (from day 1 to day 5) with MEAPR, AEAPR and indomethacin, the withdrawal thresholds were significantly increased (*p* < 0.001) compared to the day before the treatment (day 0). The interruption of the treatment induced a reduction in paw withdrawal threshold of animals treated with APR extracts at all doses. However, this weakening of the antinociception reaction after the treatment interruption was not significant at the higher dose. The significant increase of threshold responses reappeared with the resumption of treatment (**Figure 4**).

#### Acetic Acid-Induced Abdominal Writhing

**FIGURE 5:**
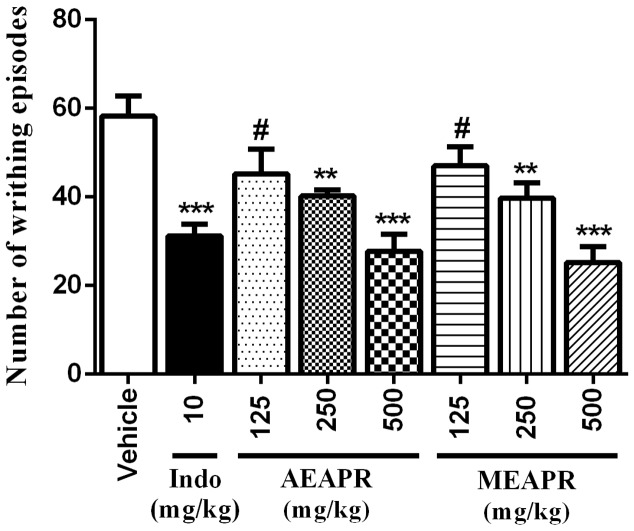
Effect of orally administrated of MEAPR and AEAPR on acetic acid-induced writhing movements in mice. Animals were treated orally with vehicle (distilled water), indomethacin (Indo), MEAPR or AEAPR (125, 250, and 500 mg/kg), prior to acetic acid injection (0.6%, i.p.). Each point represents mean ± SEM of writhes number for six mice per group. The symbols denote the significance levels: ^∗∗^*p* < 0.01 and ^∗∗∗^*p* < 0.001 in comparison with the vehicle-treated group; ^#^*p* < 0.05 in comparison with the indomethacin-treated group, using Newman–Keuls *post hoc* analysis.

The oral administration of AEAPR or MEAPR at a dose of 250 and 500 mg/kg reduced the number of writhes compared to the vehicle group (**Figure 5**). The one-way ANOVA analysis showed a significant effect of treatment on the number of writhes [*F*_(7,48)_ = 8.14, *p* < 0.001]. In fact, *post hoc* analysis showed that the extent of induced writhing at doses of 250 and 500 mg/kg of AEAPR or of MEAPR was significantly reduced compared to the vehicle group (AEAPR: *q* = 4.66, *p* < 0.01; *q* = 7.80, *p* < 0.001; and MEAPR: *q* = 4.76, *p* < 0.01; *q* = 8.55, *p* < 0.001); whereas both extracts of APR at 125 mg/kg, did not produce an antinociceptive effect (*p* > 0.05). In addition, the number of mouse abdominal constrictions induced by acetic acid in MEAPR treated group did not differ from that of the AEAPR treated group (*p* > 0.05) at high doses. Moreover, the test revealed that both extracts, at a dose of 125 mg/kg, reduced the number of writhes but to a lesser degree than that observed with indomethacin (MEAPR: *q* = 4.09 and AEAPR: *q* = 3.80, *p* < 0.05), unlike the effect of the two higher doses (250 and 500 mg/kg), which did not significantly differ from indomethacin’s one (*p* > 0.05). APR extracts treatments at 125, 250, and 500 mg / kg induced contraction inhibition of 19.2, 30.9, and 52.2% respectively for AEAPR and 22.3, 31.6, and 56.7%, respectively, for MEAPR, while the standard drug (indomethacin) had an inhibition rate of 46.4%.

#### Thermal Sensitivity

**FIGURE 6:**
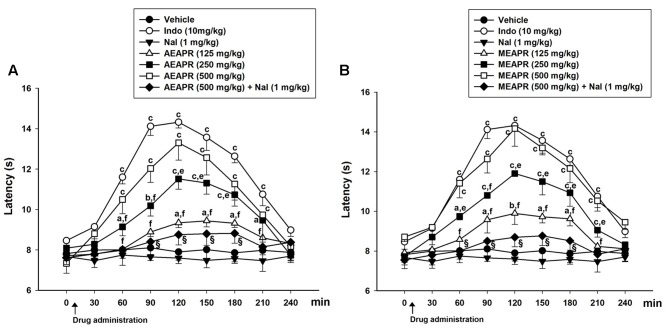
Effect of single administration of AEAPR **(A)** and MEAPR **(B)** in the hot plate test. Animals (six mice each group) were treated orally with vehicle (distilled water), indomethacin (Indo), MEAPR or AEAPR (125, 250, 500 mg/kg or 500 mg/kg + naloxone (1 mg/kg) (Nal), prior to 55°C thermal tests. Naloxone was administered alone or 30 min before administration of AEAPR and MEAPR. Each point represents the mean ± SEM of the response latency (in seconds) at different time points after the drug administration. ^a^*p* < 0.05, ^b^*p* < 0.01, and ^c^*p* < 0.001 in comparison with the vehicle-treated group; ^d^*p* < 0.05, ^e^*p* < 0.01, and ^f^*p* < 0.001 in comparison with the indomethacin-treated group, using Holm–Sidak *post hoc* analysis.

In the hot plate test, the oral administration of indomethacin, MEAPR or AEAPR significantly increased the paw withdrawal latency compared to the vehicle-treated group (**Figure 6**). This effect began 60 min after oral administration of high doses and then disappeared after 240 min. At a lower dose of APR extracts (125 mg/kg), the paw withdrawal latency started to increase significantly after 90 min of treatment with MEAPR and only after 120 min of treatment with AEAPR (**Figure 6**); two-way repeated measures ANOVA (group and time effect) confirmed these findings [*F*_(7,48)_ = 17.13, *p* < 0.001 and *F*_(7,48)_ = 69.52, *p* < 0.001, respectively]. Compared to baseline, the *post hoc* analysis showed that the treatment with indomethacin, MEAPR or AEAPR at higher doses (250 and 500 mg/kg) increased significantly the paw withdrawal latency, starting from 60 to 180 min (*p* < 0.001). In addition, naloxone (an opioid antagonist), which showed any significant effect on thermal sensitivity (in comparison with control: *t* = 0.746, *p* > 0.05), partially reversed the effect induced by AEAPR or MEAPR at 500 mg/kg in the hot plate test. In fact, we observed a decrease of the paw withdrawal latency of 33.68 or 38.77%, respectively, in comparison with both AEAPR and MEAPR at time point 120 min.

#### Formalin-Induced Pain

**FIGURE 7:**
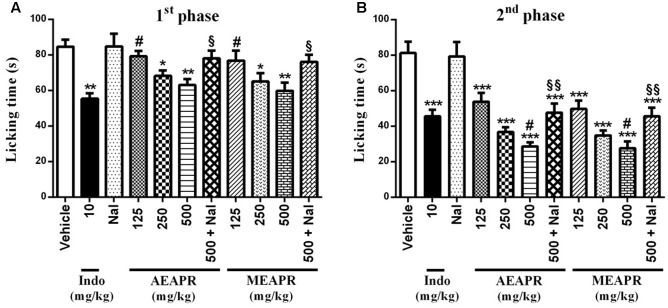
Effect of MEAPR and AEAPR on the paw licking response induced by formalin in mice. Animals were treated orally with vehicle (distilled water), indomethacin (Indo), MEAPR or AEAPR (125, 250, 500 mg/kg or 500 mg/kg + naloxone, prior to intraplantar injection of formalin (20 μl). Naloxone was administered alone or 30 min before administration of AEAPR and MEAPR. The total time spent licking the hind paw was measured **(A)** in the first phase (0–5 min) and **(B)** in the second phase (15–30 min) after intraplantar injection of formalin. Vertical bars represent mean ± SEM of licking time for six mice in each group. The symbols denote the significance levels (one-way ANOVA followed by Newman–Keuls test): ^∗^*p* < 0.05, ^∗∗^*p* < 0.01, and ^∗∗∗^*p* < 0.001 compared with the vehicle-treated group; ^#^ *p* < 0.05 compared with the indomethacin-treated group. AEAPR, aqueous extract of *Anacyclus pyrethrum* root; MEAPR, methanol extract of *Anacyclus pyrethrum* root; Indo, indomethacin; Nal, naloxone.

In the formalin test, pain responses such as licking of the right hind paw were expressed in the first and second phases. The licking time of the first phase was reduced when the mice were treated with 125, 250, and 500 mg/kg of MEAPR (**Figure 7A**), and one-way ANOVA confirmed these differences [*F*_(7,48)_ = 4.95, *p* < 0.001]. The *post hoc* analysis showed a significant difference between treated groups with 250 and 500 mg/kg of MEAPR or AEAPR and vehicle-treated group (*p* < 0.05). In addition, the analysis showed a significant difference between AEAPR at 125 mg/kg and indomethacin (*p* < 0.05), whereas there was no significant difference between indomethacin and MEAPR treated groups (*p* > 0.05). Concerning the second phase, all treatments markedly reduced the licking time of the injected paw in comparison with the group treated with vehicle (**Figure 7B**). The one-way ANOVA indicated that all treatments induced significant differences of the mean paw licking time in comparison to vehicle-treated group [*F*_(7,48)_ = 18,25, *p* < 0.001]. The *post hoc* analysis showed that the licking times at all doses of each APR extract and indomethacin were significantly (*p* < 0.001) less than that of the vehicle group. In contrast to the first phase, the effect of MEAPR and AEAPR at 500 mg/kg scored higher (*p* < 0.05) than indomethacin in the second phase with percentages of inhibition of 66, 64, and 43%, respectively. The pre-treatment with naloxone (1 mg/kg) reversed the antinociceptive activity of AEAPR and MEAPR at a dose of 500 mg/kg, i.e., an increase in the licking time of the first phase (23.80 and 27.09%; respectively) and the second phase (66.43 and 65.21%; respectively) in the formalin test. While the effect of naloxone treatment alone, did not differ from that of the vehicle (*p* > 0.05).

### Measurement of Motor Performance and Locomotor Activity

Treatment with MEAPR or AEAPR, even at the highest dose tested (500 mg/kg), did not significantly reduce locomotion in the open field (vehicle: 2772.43 ± 144.72 cm; MEAPR: 2841.30 ± 338.26 cm; AEAPR: 2948.83 ± 320.80 cm; Indo: 2947.02 ± 189.08 cm, n.s), and produced no difference in latency to fall off the bar in the rotarod test (vehicle: 281.66 ± 14.30 s; MEAPR: 274.04 ± 25.91 s; AEAPR: 279.75 ± 20.21 s; Indo: 269.70 ± 19.11 s, n.s).

### Antioxidant Activity of *Anacyclus pyrethrum* Extracts

The antioxidant activity of *Anacylcus pyrethrum root* was assessed by three complementary *in vitro* antioxidant assays: the DPPH, the FRAP and the BCB assays. The concentrations that led to 50% inhibition (IC_50_) are given in **Table 2**. Note that low IC_50_ values reflect better protective action. The results showed that both *Anacylcus pyrethrum* extracts (MEAPR and AEAPR) exhibited similar and interesting antioxidant activity, especially in DPPH test with IC_50_ values of 12.38 ± 0.28 μg/ml and 13.41 ± 0.67 μg/ml respectively. The antioxidant potencies of both extracts were significantly less than those of the reference antioxidants butylated hydroxytoluene (BHT) and quercetin (**Table 2**). No significant difference was observed between AEAPR and MEAPR in any of the three antioxidant assays (*p* > 0.05). Recorded results showed that among the used bio-assays, the antioxidant activities of MEAPR and AEAPR were significantly higher, with DPPH than with other assays (*p* < 0.001).

**Table 2:** Antioxidant activities, expressed as IC50 values using DPPH, FRAP and BCB assays, for AEAPR, MEAPR, BHT and quercetin.

Sample Extracts	Test		
	DPPH (μg/ml)	FRAP (μg/ml)	BCB (μg/ml)
MEAPR	12.38 ± 0.25^∗∗∗^^###^	50.89 ± 1.25^∗∗∗^^###^	107.07 ± 4.14^∗∗∗^^###^
AEAPR	13.41 ± 0.67^∗∗∗^^###^	60.17 ± 4.48^∗∗∗^^###^	120.66 ± 3.61^∗∗∗^^###^
BHT	4.21 ± 0.08	7.09 ± 0.10	4.30 ± 0.33
Quercetin	1.07 ± 0.01	2.29 ± 0.10	0.95 ± 0.02

*Values are the mean of three determinations ± SEM, ^∗∗∗^*p* < 0.001 significantly different from the BHT group and ^###^*p* < 0.001 significantly different from the quercetin group. DPPH, 2,2-diphenyl-1-picrylhydrazyl; FRAP, ferric reducing antioxidant power; BCB, β-carotene bleaching; BHT, butylated hydroxy toluene*.

## Discussion

In the present work, APR extracts were first investigated for acute toxicity in male Swiss mice. AEAPR and MEAPR did not produce any sign of acute toxicity nor mortality up to the maximum dose of 5000 mg/kg during the 14 days following their single administration. Thus, both extracts have an LD_50_ higher than 5000 mg/kg. According to the chemical labeling and classification of acute systemic toxicity recommended by OECD, AEAPR and MEAPR were assigned to the lowest toxicity class (OECD, 2001), which is in accord with the conclusions of previous studies (Doudach et al., 2012; Kishor Kumar and Lalitha, 2013).

In the present study, the evaluation of the activity of MEAPR and AEAPR revealed that both extracts possessed potent anti-inflammatory effects in both acute and chronic models of inflammation. Indeed, we have shown that that AEAPR and MEAPR exhibited higher activity to counter the acute inflammation in a xylene-induced-ear-edema model (65 and 62%; respectively) than that of indomethacin (49%). This model is widely used to evaluate anti-inflammatory topical steroids and non-steroidal anti-inflammatory agents, especially those that inhibit phospholipase A2 (Zanini et al., 1992; Núñez Guillén et al., 1997). In addition, several studies have shown that the xylene increases vascular permeability, which causes edema that indicates inflammation, due to the release of inflammatory agents such as bradykinin, prostaglandin, histamine and serotonin, which in turn release neuropeptides that activate their receptors, producing neurogenic inflammation (Carlson et al., 1985; Banki et al., 2014). Substance P is one of these neuropeptides, which allows the release of nitric oxide from endothelial cells, triggering vasodilation and plasma exudation, which is the origin of edema formation (Barry et al., 2011). On the basis of these data, the significant inhibition of xylene-induced ear swelling by AEAPR and MEAPR is certainly due to the action of the phytochemicals detected in those extracts and which may act, individually or in synergy, at different levels of the multifactorial process of inflammation. Among the potential active metabolites flavonoids have a membrane-stabilizing effect by reducing vasodilatation, which ameliorates the strength and integrity of blood vessel walls (Pathak et al., 1991); while alkaloids may act through the prevention of the neurogenic inflammation.

In addition, to investigate the effects of APR in a sustained inflammation model, we evaluated the effect of AEAPR and MEAPR on inflammation induced by intraplantar injection of CFA. Our results showed for the first time that acute or chronic oral treatments of animals with MEAPR or AEAPR were effective in preventing not only paw edema caused by CFA injection, but also mechanical hypersensitivity. Moreover, the anti-edematogenic and antinociceptive actions of APR extracts were evident from an early stage (1 h30) and maintained up to 7 h.

It has been reported that CFA induces persistent pain that results mainly from the involvement of macrophages and T lymphocytes in the injected rat paw, followed by a paw swelling and leukocyte infiltration of the synovium and surrounding tissue, which contribute to chronic inflammation and osteolytic lesions (Butler et al., 1992; Romas et al., 2002; Mo et al., 2013). In addition, activation of macrophages results in the production of pro-inflammatory cytokines (IL-1β and TNF-α), growth factors and other inflammatory mediators, resulting in peripheral sensitization (Kumar et al., 2010; Mo et al., 2013). Indeed, inflammation causes the induction of COX-2 (Vane and Botting, 1998) leading to the release of nitric oxide, bradykinin and prostanoids, which induce phosphorylation of ion channels in nociceptor terminals, enhancing excitability and reducing the nociception threshold, and therefore development of peripheral sensitization. This mechanism of nociceptors sensitization is widely involved in all types of inflammatory pain and is associated with chronic pain (Basbaum, 1999; Basbaum et al., 2009).

Since the antinociceptive effect of AEAPR and MEAPR is associated with anti-inflammatory action, its continuing antinociceptive effect in the chronic pain model may be due to a reduction in the cytokine and prostanoid release which reduced sensitization of the nociceptors. It should be noted that Crombie (1954) has isolated dodeca-2E,4E-dienoic acid isobutylamide from APR (i.e., pellitorine) and that Cech et al. (2010) have reported that the same extract component from *Echinacea purpurea* roots exerted immuno-modulatory effects, especially with a decrease of plasma protein levels of certain pro-inflammatory cytokines (IL-8 and IL-6) and inversely an increased expression of anti-inflammatory molecules such as IL-10. Thus, although we didn’t quantify the levels of the cytokines, we may predict that APR extract may act via the same product (dodeca-2E,4E-dienoic acid isobutylamide) to blunt the induced inflammation.

Assessment of AEAPR and MEAPR effects on the abdominal constrictions elicited by acetic acid showed a marked suppression of writhing response in the visceral pain model. This test is mainly used to screen antinociceptive activity (Utsunomiya et al., 1998). It has been reported that acetic acid induces the release of endogenous mediators that activate the nociceptive neurons (Collier et al., 1968). Indeed, acetic acid acts by releasing biogenic amines (e.g., bradykinin, and serotonin), cyclooxygenases and their metabolites (e.g., PGE2 and PGF2α) in the peritoneal fluid (Derart et al., 1980; Dhara et al., 2000). It also activates peritoneal receptors (Bentley et al., 1983; Medzhitov, 2008) and stimulates nociceptive nerve terminals (Duarte et al., 1988). The present work demonstrated that indomethacin causes a significant inhibition of acetic acid-induced pain, which is in agreement with previous reports indicating that this test is sensitive to non-steroidal anti-inflammatory drugs (NSAIDs) (Gené et al., 1998; Vane and Botting, 2003). According to Rimbau et al. (1999), the alkamides from APR extracts act as a dual inhibitor of cyclooxygenase (COX) and 5-lipoxygenase (LOX) enzymes. COX catalyzes the conversion of arachidonic acid to prostaglandin (Williams et al., 1999), leading to the activation and sensitization of peripheral nociceptors. Since the vast majority of studies of alkamides shows their peripheral antinociception effects (de la Rosa-Lugo et al., 2017), on the basis of the Rimbau et al. (1999) study and our current findings, we suggest that the peripheral anti-nociception activity of AEAPR and MEAPR may be due to their alkamides.

The central antinociceptive activity of MEAPR and AEAPR was evaluated in the hot plate test. This test is a widely used model for acute thermal nociception to evaluate specifically central nociception (Eddy and Leimbach, 1953). Through this test of complex responses to inflammation and nociception (Bhandare et al., 2010), centrally acting antinociceptive drugs (i.e., opioid agents) elevate the nociception threshold of rodents toward heat via spinal and supraspinal receptors (Zakaria et al., 2008; Amabeoku and Kabatende, 2012). The present results showed that the oral treatment with both extracts of APR provided antinociceptive effects in a dose-dependent manner, indicating likely a central action. Furthermore, treatment with indomethacin (an NSAID drug) induced a significant increase in the latency time in the hot plate test, which is in accordance with a previous study indicating that the NSAID shows a central anti-nociception mediated by either serotoninergic (5-HT2/5-HT3) or adrenergic (α1/α2) receptors at the spinal/supraspinal level (Arslan and Bektas, 2015). Therefore, the efficacy of AEAPR and MEAPR in the hot plate test might be due to analgesic agent(s) acting primarily at the spinal, medullary, and/or higher levels of the CNS or by some indirect mechanisms as suggested for narcotic substances by Yaksh and Rudy (1977) and Tjølsen et al. (1992). Otherwise, it has been reported that the antinociceptive effect of affinin, alkamides isolated from Heliopsis longipes, could be a result of the activation of opioidergic, serotoninergic and GABAergic systems (Déciga-Campos et al., 2010). Indeed, the most important phytoconstituents present in the APR are alkamides (Crombie, 1954; Boonen et al., 2012). Thus, the central anti-nociception of MEAPR and AEAPR may due to its alkamides component.

To discriminate between the peripheral and central antinociceptive effects of APR extracts, the formalin test was used. Our results revealed that both AEAPR and MEAPR have acted effectively in both phases of the formalin test. It is known that the intraplantar injection of formalin induces two phases of pain sensitivity (Tjølsen et al., 1992). The first and the second phases are characterized as neurogenic pain and inflammatory pain, respectively. The mechanisms underlying these two phases are previously reported (Hunskaar and Hole, 1987; Corrêa and Calixto, 1993; do Amaral et al., 2007; McNamara et al., 2007). Based on previous literature, we believe that our results show MEAPR and AEAPR exert their analgesic action at both central and peripheral levels.

Moreover, in this study, we demonstrated that the antinociceptive action of AEAPR and MEAPR was partially antagonized by naloxone in the hot plate, and in the formalin test (first and the second phase). These results suggest that the APR extracts mechanism involves in part opioid receptors.

In addition to this, we did not detect any disturbances in the locomotor activity or motor performance in animals treated by AEAPR or MEAPR up to 500 mg/kg. This suggests that both extracts at the highest effective dose, have no muscle-relaxant or central depressant action in models of nociception used in our study. Our result is in opposition of the study of Zaidi et al. (2013), which demonstrated that ethanolic extract of APR impaired motor coordination at a dose of 1600 mg/kg in Rotarod performance. However, this difference could be due to environmental conditions which could influence the expression of phytochemical compounds in the same plants grown in different areas (Harborne, 1993; Lee et al., 2007). In addition, several studies have reported variations in the biological activities of extracts prepared using different extraction techniques (Dhanani et al., 2017).

Inflammation is a process that involves a series of phenomena that may be due to several agents. It is usually associated with pain which is a secondary phase resulting from the release of analgesic mediators (Tsai et al., 2001). The inflammatory reaction could also be initiated by oxidative stress, which is defined as an overproduction of oxidizing molecules namely ROS. These agents induce cytokine release and pro-inflammatory enzyme activation; which are involved in the inflammatory process (Gupta et al., 2005). Indeed, inflammation, pain and oxidative stress are interrelated processes. In the present study, AEAPR and MEAPR showed an anti-inflammatory activity, therefore, both extracts of *Anacyclus pyrethrum* root were investigated for their antioxidant action against free radicals using diverse methods (DPPH, FRAP and BCB). The results showed that APR extracts, according to DPPH method, have a strong scavenging activity, but with the capacity to protect against lipid peroxidation (BCB test). Indeed, *Anacyclus pyrethrum* effectively inhibits oxidative stress, which is in accordance with previous studies (Sujith et al., 2011a; Selles et al., 2012b).

The antioxidant potential of APR extracts may be due to their phytochemical constituents. In fact, phytochemical screening has proved the occurrence of alkaloids, flavonoids, saponins, tannins, triterpenes and sterols. Detection of these phytochemicals in APR agrees with earlier findings (Sujith et al., 2011a; Selles et al., 2012a; Hanane et al., 2014). The phenolic compounds may contribute directly to the antioxidative action as has been shown by Nagendrappa (2005). Generally, flavonoids, which have powerful antioxidant activities *in vitro*, are able to scavenge a wide range of ROS like superoxide and nitric oxide radicals (Halliwell, 2008). In addition, as opioid receptors have been partially involved in APR extract’s effects, detected alkaloids may be among the active compounds of APR extracts.

## Conclusion

Our study demonstrated that aqueous and methanol extracts of *Anacyclus pyrethrum* roots are non-toxic substances, with good central and peripheral antinociceptive effects, which is beneficial and researched in traditional medicine. The qualitative phytochemical analysis and the antioxidant activity *in vitro* have revealed the presence of several antioxidant phytoconstituants such as flavonoids, alkamides, saponins and tannins in Anacyclus pyrethrum root extracts.

Many mechanisms of specific action to those phytochemical compounds could be responsible for anti-inflammatory and antinociceptive activities observed in the present work. However, further studies are needed to isolate the pharmacologically active compounds and elucidate their exact molecular mechanism in the anti-inflammatory and antinociceptive process of APR.

## Author Contributions

HM, MB, ZS, and SB designed the experiments; HM and OB performed the experiments, HM, SB, MB, and ACG performed the analysis of the data; HM And SB assembled the figures. HM, SB, MB, and ACG wrote and edited the manuscript. All authors validated it.

## Conflict of Interest Statement

The authors declare that the research was conducted in the absence of any commercial or financial relationships that could be construed as a potential conflict of interest.

## Abbreviations

NHV: AEAPR
Aqueous Extract of *Anacyclus pyrethrum* Roots: BCB
β-carotene bleaching: BHT
butylated hydroxy toluene: CFA
Complete Freund’s adjuvant: COX
cyclooxygenase: DPPH
2: 2-di-phenyl-1-Picryl-Hydrazyl
FRAP: ferric reducing antioxidant power
i.p.: intraperitoneal administration
Indo: indomethacin
LOX: 5-lipoxygenase
MEAPR: Methanol Extract of *Anacyclus pyrethrum* Roots
NSAIDs: non-steroidal anti-inflammatory drugs
ROS: reactive oxygen species.
